# Selective Root Canal Retreatment: A Scoping Review and Metasynthesis by Thematic Analysis

**DOI:** 10.1111/aej.70050

**Published:** 2026-01-02

**Authors:** José Evando da Silva‐Filho, Júlia Magalhães‐Saldanha, Ana Paula Caracas‐de‐Araújo, Zildenilson da Silva Sousa, Danielle Frota de Albuquerque, Eduardo Diogo Gurgel‐Filho

**Affiliations:** ^1^ Department of Dental Radiology and Imaging, Faculty of Dentistry University of Fortaleza Fortaleza Ceará Brazil; ^2^ Department of Endodontics, Faculty of Dentistry University of Fortaleza Fortaleza Ceará Brazil; ^3^ Department of Operative Dentistry, Faculty of Dentistry University of Fortaleza Fortaleza Ceará Brazil; ^4^ Department of Stomatology, Faculty of Dentistry University of Fortaleza Fortaleza Ceará Brazil

**Keywords:** cone‐beam computed tomography, minimally invasive dentistry, scoping review, selective root canal retreatment, thematic analysis

## Abstract

Selective root canal retreatment preserves well‐filled canals by treating only roots showing clinical or imaging evidence of failure, providing a minimally invasive alternative to conventional full retreatment. This scoping review, supported by metasynthesis through thematic analysis, synthesised clinical and imaging evidence in multirooted teeth. Five studies, including experimental research and case reports, were analysed. Thematic analysis revealed four core themes: clinical outcomes, imaging assessment, procedural features, and methodological limitations. Short‐term outcomes of selective retreatment were comparable to full retreatment, with high tooth survival and low incidence of new lesions. Three‐dimensional imaging played a pivotal role in detecting root‐confined pathology, planning selective access, and supporting structural preservation. Evidence remains limited by small sample sizes, retrospective designs, and heterogeneity in study protocols. This review offers a structured synthesis of selective retreatment, integrating clinical and imaging insights to inform minimally invasive endodontic practice in a research and clinical context.

## 1 Introduction

Endodontic treatment aims to achieve maximum disinfection of the root canal system. Unsuccesful outcomes may result from several factors, including inadequate mechanical debridement, persistence of bacteria in the canal system or apical region, poor‐quality obturation, overextension or underfilling of canals, and deficient coronal sealing [1, 2, 3]. Conventional endodontic retreatment is traditionally considered the standard approach in cases of treatment failure.

Advancements in imaging have improved the evaluation of endodontic cases. The widespread use of cone‐beam computed tomography (CBCT), with its three‐dimensional capabilities, enables precise and detailed analysis of periapical conditions. This technological evolution has driven updates in clinical dental practice. It may enable selective root canal retreatment (SRCR) as a therapeutic option for previously treated teeth presenting with post‐treatment disease [4, 5].

In this context, SRCR has been proposed as an approach that could preserve adequately filled canals and intervene only in those with clinical or imaging evidence of failure. The procedure may be limited to a single root or to those showing periapical pathology, while maintaining the integrity of roots without clinical or radiographic signs of involvement [6, 7].

This minimally invasive approach appears to reduce damage to dental structures, lower the risk of iatrogenic complications, and preserve apical sealing integrity [8]. Nevertheless, despite increasing interest in SRCR, there is still a scarcity of systematic, controlled, and randomised studies that confirm its effectiveness, establish eligibility criteria, and evaluate long‐term outcomes.

These gaps hinder the indication of SRCR and the standardisation of clinical protocols, generating uncertainty regarding their efficacy. Therefore, this article aims to map, through a scoping review with metasynthesis by thematic analysis, the clinical and imaging foundations that may support the decision for SRCR.

## 2 Material and Methods

### 2.1 Protocol and Registration

This scoping review was conducted following the Joanna Briggs Institute methodology for scoping reviews [9]. The metasynthesis was performed through thematic analysis in accordance with the framework proposed by Braun and Clarke [10]. The manuscript was prepared following the Preferred Reporting Items for Systematic Reviews and Meta‐Analyses extension for Scoping Reviews (PRISMA‐ScR) guidelines [11]. The study protocol was registered on the Open Science Framework (OSF) and assigned the following identifier: 10.17605/OSF.IO/NDSCJ

### 2.2 Research Question

The Population–Concept–Context (PCC) framework was adopted to formulate the guiding question: “What are the clinical and imaging‐based foundations that support decision‐making in selective endodontic retreatment of previously treated teeth?” (Table 1).

**TABLE 1:** Structured PCC framework for guiding question construction.

C	D	S
P	Population	Previously endodontically treated teeth
C	Concept	Selective endodontic retreatment approach
C	Context	The clinical and imaging‐based criteria for treatment planning

Abbreviations: C, Component; D, Definition; S, Specification.

### 2.3 Data Sources and Search Strategies

Comprehensive searches were conducted to identify the maximum available evidence regarding the SRCR. Search strategies were developed combining Medical Subject Headings terms with related keywords using the Boolean operators “AND” and “OR” (Table 2). These strategies were implemented on 18 April 2025 using advanced search tools across the following databases: Cochrane Library, Embase, PubMed, Scopus, ScienceDirect, Virtual Health Library, and Web of Science. No filters or limits were applied. The searches were continuously updated and reviewed from April through August 2025 to ensure completeness.

**TABLE 2:** Databases and adapted search strategies.

DATABASE	STRATEGY
Cochrane Library	(“Endodontics” OR “Root Canal Therapy”) AND “Retreatment” AND (selective OR partial)
Embase	(‘endodontics’/exp. OR ‘root canal therapy’/exp) AND ‘retreatment’/exp. AND (selective:ti,ab OR partial:ti,ab)
PubMed	((“Endodontics”[Mesh] OR “Root Canal Therapy”[Mesh]) AND “Retreatment”[Mesh]) AND (“Selective”[Title/Abstract] OR “Partial”[Title/Abstract])
Scopus	TITLE‐ABS‐KEY ((“endodontic retreatment” OR “root canal retreatment”) AND (selective OR partial))
ScienceDirect	(“endodontic retreatment” OR “root canal retreatment”) AND (selective OR partial)
Virtual Health Library	(“Endodontia” OR “Tratamento do Canal Radicular” OR “Endodontics” OR “Root Canal Treatment”) AND (Retratamento OR “Retratamento Endodôntico” OR Retreatment OR “Endodontic Retreatment”) AND (Seletiv* OR Parcial* OR Selectiv* OR Partial*)
Web of Science	TS = (“endodontic retreatment” OR “root canal retreatment”) AND TS = (selective OR partial)

### 2.4 Eligibility Criteria

Eligible studies reported clinical or imaging outcomes of SRCR, regardless of study design, population, follow‐up, or laboratory and ex vivo studies. Studies limited to conventional full retreatment or animal models were not considered.

### 2.5 Study Selection

Study selection was completed by two previously calibrated independent authors in two phases. The first phase consisted of reading titles and abstracts during the screening process; studies that appeared to meet the eligibility criteria were selected for the second phase, in which full texts were independently read by the same two authors, and the eligibility criteria were definitively applied after duplicate removal. Disagreements between the two reviewers were resolved by the supervisors of this research after the blinding phase ended.

### 2.6 Data Extraction

Studies were organised into Google Sheets (docs.google.com/spreadsheets/) by a single author and categorised according to the following variables: (i) Experimental Studies—authorship, year and country of origin, title, objective(s), study design, number of participants, eligibility criteria, methods, main results, and conclusion; (ii) Case Reports—authorship, year and country of origin, title, case description, imaging findings, protocol used, follow‐up duration, and conclusions.

### 2.7 Measured Outcomes

The primary outcome of this review was to evaluate the clinical and imaging foundations supporting SRCR. This was assessed through success rates and changes in periapical lesions. Secondary outcomes included the influence of imaging techniques, particularly CBCT, on treatment decisions, functional improvement, and follow‐up duration.

### 2.8 Metasynthesis by Thematic Analysis

The metasynthesis by thematic analysis was conducted qualitatively using findings from the scoping review. Following Braun and Clarke's framework [10], six sequential steps were applied: (i) familiarisation with the data through repeated reading and noting initial impressions; (ii) generating initial codes to identify relevant features; (iii) searching for themes by collating related codes; (iv) reviewing themes for coherence and clear distinctions; (v) defining and naming themes to capture their essence; and (vi) producing the final report, integrating examples, interpretation, and links to the research question. Data were compared and feasibility assessed, with SRCR contrasted against conventional retreatment and imaging roles analysed. Comparisons were structured along three axes: clinical outcomes, radiographic characteristics, and procedural features, with heterogeneity and evidence quality considered throughout.

## 3 Results

**FIGURE 1:**
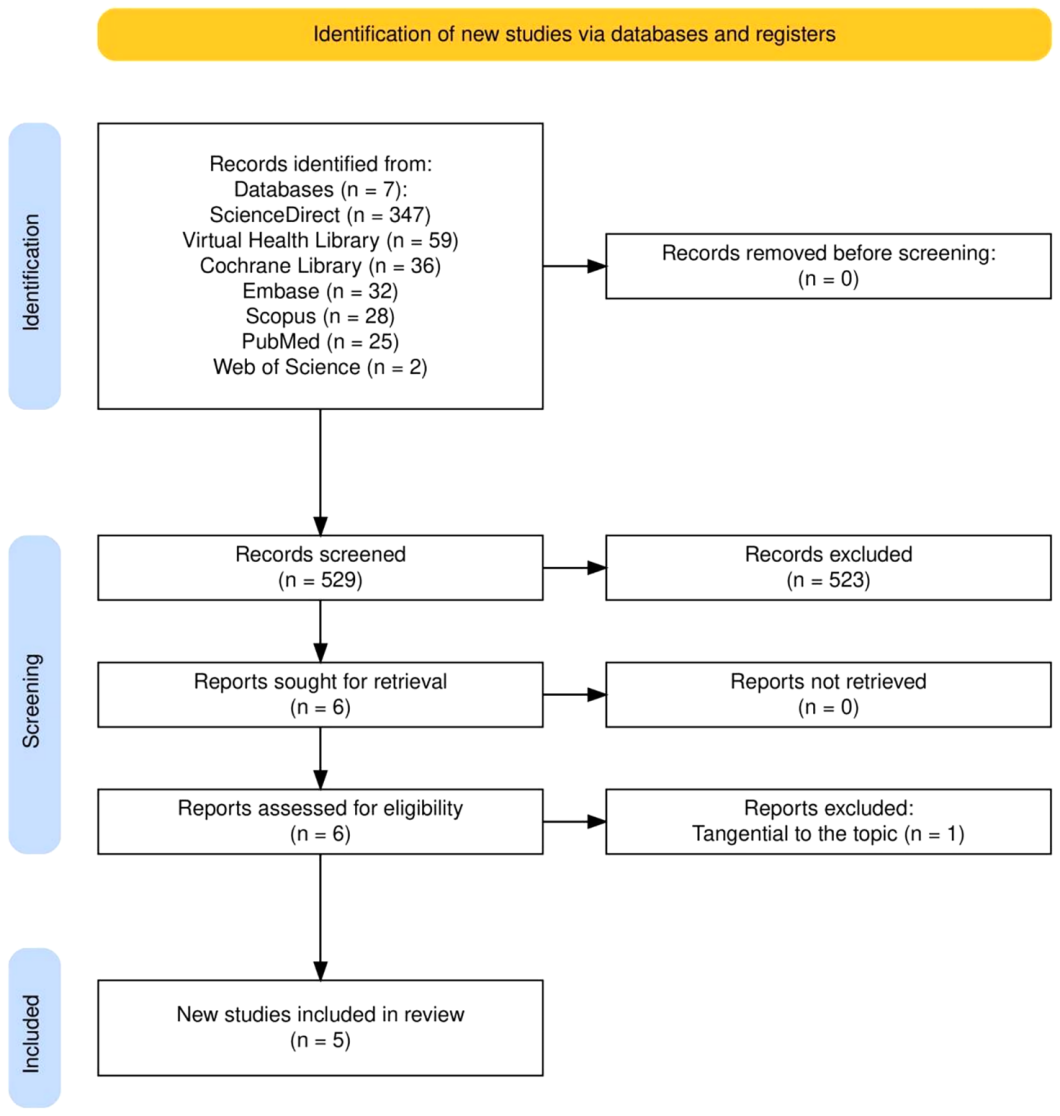
PRISMA‐based flowchart for study selection.

A total of 529 studies were retrieved across seven databases. After title and abstract screening by two independent researchers, 17 studies were selected for further review. Following the removal of 11 duplicates, 6 studies were assessed in full text. After applying the eligibility criteria, 5 studies were included in this scoping review and metasynthesis. The study selection process is charted in Figure 1. Details regarding the databases, search strategies, total results, and the studies selected and included were combined and are available in File S1.

### 3.1 Overview *of Included Studies*

The selected studies focus on SRCR and describe the technical procedures and assess clinical effectiveness, reporting favourable outcomes. CBCT is highlighted as a valuable tool for accurate diagnosis, treatment planning, and follow‐up of selective retreatment. The experimental studies are summarised in Table 3 and in detail in File S2; case reports are summarised in Table 4.

**TABLE 3:** Overview of experimental studies.

Authorship, year and origin	Title	Study design	Objective	Design/sample (N)	Eligibility	Methods	Key results
Brochado‐Martins et al., 2022, Netherlands	Outcome of selective root canal retreatment—A retrospective study	Retrospective study	To assess (i) clinical and radiographic/CBCT outcomes of SRCR (≥ 12 months), (ii) periapical status of untreated roots, (iii) tooth survival	Retrospective; 75 pts.; 75 teeth (195 roots)	Incl: multirooted teeth, symptomatic AP, ≥ 1 untreated root, healthy periodontium, intact restoration. Excl: retreatment of all canals, VRF, prior surgery, no f/u imaging	SRCR by certified endodontists under RD + DOM. Follow‐up via PR or CBCT. Outcomes rated [1, 2, 3, 4, 5, 6]; lowest root score defined tooth‐level result. Statistical tests: Fisher's exact, κ	92.7% favourable in retreated roots; 3.5% new lesions in untreated. Overall tooth success: 86.7%. No prognostic factor significant. Tooth survival: 91.5%. Intraobserver agreement: PR 0.91; CBCT 0.88
Nagy; Ghobashy, 2022, Egypt	Selective Root Retreatment: A Randomised Clinical Trial	Randomised, controlled, double‐blind, multicenter clinical trial with a parallel‐group design included	To compare SRCR vs. traditional retreatment prognosis	RCT, double‐blind, multicenter; 40 pts	Incl: ≥ 1 root with PAI ≥ 3, intact restoration. Excl: systemic disease, multiroot lesions, fractures	Groups: SRCR vs. full retreatment. CBCT + DOM, ProTaper Next, NaOCl, CW obturation. PAI measured at 3, 6, 9, 12 months; CBCT at 12 month	No significant difference in success (PAI) at 12 month (*p* = 0.853)
Turky; Elfatah; Hamdy, 2024, Egypt	Does selective root canal retreatment preserve the tooth's fracture resistance? An ex vivo study	Ex Vivo Study	To compare fracture resistance in SRCR vs. conventional retreatment	Ex vivo; 33 extracted mandibular molars	Incl: mature roots, canal curvature < 20°, no cracks/restorations. Excl: caries, resorptions, non‐negotiable canals	MOD prep + SRCR or full retreatment with HyFlex CM. Bioceramic sealer. Aging simulation. Load test on Instron. Failure type classified	SRCR group showed higher fracture resistance (1106.8 ± 159.8 N) vs. full retreatment (867.7 ± 108.9 N) (*p* = 0.012). Non‐restorable fractures: SRCR 36.4%, full 54.5%

Abbreviations: AP, Apical Periodontitis; CBCT, Cone‐Beam Computed Tomography; CW, Continuous Wave; DOM, Dental Operating Microscope; f/u, Follow‐Up; HyFlex CM, Controlled Memory Nickel‐Titanium Rotary System; Instron, Universal Testing Machine (Instron Corp.); MOD, Mesio‐Occluso‐Distal; N, Sample Size; NaOCl, Sodium Hypochlorite; PAI, Periapical Index; PR, Periapical Radiograph; pts., Patients; RCT, Randomised Controlled Trial; RD, Rubber Dam; SRCR, Selective Root Canal Retreatment; VRF, Vertical Root Fracture; WL, Working Length; κ, Cohen's Kappa.

**TABLE 4:** Overview of case reports.

Authorship, year and origin	Title	Case description	Imaging	Protocol	Follow‐up	Conclusion
Nudera, 2015, United States of America	Selective Root Retreatment: A Novel Approach	57‐y/o F; localised pain (2 week); tooth #18 (ADA): tender to palpation, percussion, and biting; full crown; RPD abutment; mild gingival inflammation; normal PD	PR and CBCT: previous RCT; cast post/core; untreated DL root (RE) with apical lesion; mesial roots normal; DB root inconclusive	Profound LA and RD; access through cast core under DOM; DL canal located, WL established; prepared to 25/.06 (Vortex); irrigated with NaOCl + QMix (sonic + ultrasonic activation); obturated with CW; restored	12 month	SRCR is a viable option for managing post‐treatment disease; 3D imaging enhances diagnosis; additional roots may need treatment if symptoms persist; long‐term outcomes require further study
Guerreiro‐Viegas; Santos, 2024, Portugal/Netherlands*	Selective root canal retreatment of a maxillary first molar: a case report with a 9‐year follow up	59‐y/o M; mild discomfort on biting; tooth #16 (FDI): previous RCT, bridge abutment; clinical: – cold test, + percussion, no swelling, normal PD; composite restoration	Post/core in DB and P roots; radiolucency between post/gutta; MB root with broken file and untreated MB2; DB/P with no apical pathology	Selective retreatment of MB root; file removed; MB2 located, prepared, obturated (X2, NaOCl/EDTA, ultrasonic activation); restored with composite	9 year	SRCR is an emerging alternative to full retreatment; early outcomes are promising; untreated roots may pose disease risk; long‐term efficacy remains uncertain; RCTs needed for validation and guidance

Abbreviations: 3D, Three‐Dimensional; ADA, American Dental Association (tooth numbering system); CBCT, Cone‐Beam Computed Tomography; CW, Continuous Wave; DB, Distobuccal; DL, Distolingual; DOM, Dental Operating Microscope; EDTA, Ethylenediaminetetraacetic Acid; FDI, Fédération Dentaire Internationale (tooth numbering system); F, Female; LA, Local Anaesthesia; MB, Mesiobuccal; MB2, Second Mesiobuccal Canal; NaOCl, Sodium Hypochlorite; P, Palatal; PD, Probing Depth; PR, Periapical Radiograph; RCT, Root Canal Treatment; RD, Rubber Dam; RE, Radix Entomolaris; RPD, Removable Partial Denture; SRCR, Selective Root Canal Retreatment; WL, Working Length; X2, File from ProTaper Universal/Next system (#25/.06); y/o, Years Old.

* It is not directly stated.

### 3.2 Metasynthesis by Thematic Analysis

The metasynthesis was conducted by integrating the findings of the scoping review (*n* = 5), following the framework proposed by Braun and Clarke [10]. The process involved comparing results, assessing data feasibility, contrasting SRCR with conventional retreatment, and analysing the role of imaging techniques. The comparison was structured along three axes: clinical outcomes, radiographic features, and procedural characteristics.

All included studies were read in full. The sample comprised two clinical studies, two case reports, and one ex vivo study. Notes were taken on outcomes, imaging findings, procedural methods, and study limitations. Recurrent patterns and preliminary observations were identified. Descriptive codes were generated based on the extracted data. These included clinical outcomes such as success rates, new lesion incidence, and tooth survival; radiographic findings including periapical pathology detection, the role of CBCT, and follow‐up observations; procedural characteristics such as selective tissue removal, structural preservation, and fracture resistance; the role of imaging techniques with emphasis on diagnostic advantages of CBCT over conventional radiographs; and study limitations including small sample sizes, absence of control groups, and retrospective designs.

Codes were then grouped into broader themes that captured the main findings. Four themes were identified: (1) clinical performance of SRCR; (2) imaging assessment; (3) procedural features; and (4) methodological limitations and evidence gaps. Themes were reviewed and discussed for internal coherence and distinction. Patterns were consistent across studies, confirming the validity of the final four themes. The final themes, their titles and conclusions are summarised in Table 5.

**TABLE 5:** Thematic analysis: Identified themes, titles and key conclusions.

Theme	Title	Conclusion
1	Clinical performance of SRCR	Short‐term success appears comparable to conventional retreatment. Tooth survival seems high, and the incidence of new lesions appears low
2	Radiographic assessment and CBCT use	CBCT may enable accurate diagnosis, selective access planning, and follow‐up. It appears to identify pathologies not visible on conventional radiographs
3	Procedural features	Selective removal may preserve dental structure and increase fracture resistance, potentially reducing iatrogenic damage
4	Methodological limitations and evidence gaps	Small samples, retrospective designs, and lack of control groups limit current evidence. Prospective, multicentre studies with long‐term follow‐up are needed

Abbreviations: CBCT, Cone‐Beam Computed Tomography; SRCR, Selective Root Canal Retreatment.

### 3.3 Clinical Performance of SRCR

The two included clinical studies suggest that SRCR is comparable to conventional retreatment in terms of short‐term success. Brochado‐Martins et al. [12] reported a favourable outcome of cases treated with SRCR (86.7%), with a low incidence of new lesions (3.5%) in untreated roots and a high tooth survival rate (91.5%). Nagy and Ghobashy [13] found no statistically significant difference in healing rates between the SRCR and conventional retreatment approaches after 12 months (*p* = 0.853).

### 3.4 Imaging Assessment

Radiographic evaluation played a critical role [6, 7, 12, 13]. CBCT was essential for identifying periapical pathologies confined to specific roots, guiding selective access planning, and follow‐up. Both case reports emphasised the unique diagnostic advantages of this modality, which enabled the detection of root‐confined pathologies that were not evident on conventional periapical radiographs.

### 3.5 Procedural Features

Selective structure removal favours SRCR in terms of structural preservation. The ex vivo study by Turky et al. [8] demonstrated a statistically significant increase (*p* = 0.012) in fracture resistance in teeth treated with SRCR (1106.8 ± 159.8 N) compared to full retreatment (867.7 ± 108.9 N), reinforcing the potential biomechanical advantage of this approach.

### 3.6 Methodological Limitations and Evidence Gaps

This qualitative analysis revealed high methodological heterogeneity, particularly regarding sample size, follow‐up duration, and study design. Only one randomised clinical trial was identified in extensive searches, limiting the strength of the evidence. Case reports are illustrative but not generalisable. The retrospective design of one study and the in vitro nature of another further restrict external validity. The current body of evidence lacks the robustness required for a generalised clinical recommendation [6, 7, 8, 12, 13].

### 3.7 Integrated Analysis

The metasynthesis demonstrates that SRCR achieves short‐term success comparable to conventional retreatment, with high tooth survival and low incidence of new lesions. CBCT is critical for identifying root‐confined pathologies and guiding selective access. Selective removal preserves dental structure and improves fracture resistance. Current evidence is limited by small sample sizes, retrospective designs, and lack of control groups, highlighting the need for prospective, multicentre studies with long‐term follow‐up. Therefore, these findings should be interpreted cautiously [6, 7, 8, 12, 13].

## 4 Discussion

It is important to state that this review does not recommend SRCR as a standard protocol, given its entirely experimental nature. It provides theoretical foundations from the literature to guide clinicians and researchers. Due to the lack of high‐quality evidence, SRCR remains an experimental approach.

SRCR differs from conventional retreatment by targeting only roots with radiographic evidence of disease, preserving well‐filled canals, and adopting a minimally invasive strategy aligned with conservative dentistry principles [6, 7, 8, 12, 13]. Traditional full retreatment involves removing all restorations, possible posts, and canal filling material, regardless of periapical pathology. Despite the limited number of studies, this review formalises early discussion on SRCR, which may offer substantial clinical benefits if long‐term effectiveness is confirmed.

Decision‐making should consider patient symptoms, treatment history, and the condition of existing restorations, while procedural success depends on rigorous follow‐up, patient compliance, and the clinician's ability to identify and eliminate the cause of failure [6, 12, 13]. Clinical outcomes are encouraging: Brochado‐Martins et al. [12] reported 91.5% tooth survival, with post‐treatment extractions unrelated to SRCR, while Guerreiro‐Viegas and Santos [7] observed complete healing of affected roots and absence of symptoms in untreated roots over 9 years. However, it is crucial to stress that single cases cannot be interpreted in isolation.

Taken together, these findings suggest that short‐term success appears comparable to conventional retreatment, with high tooth survival and low incidence of new lesions. Variations in microbial virulence, patient immune status, and systemic conditions such as diabetes or autoimmune disorders may influence outcomes, emphasising the need for caution in medically compromised patients [3, 14, 15, 16].

Preservation of marginal integrity, avoidance of recurrent caries, conservative access, and thorough CBCT evaluation are critical for success [6, 17]. The restorative strategy post‐treatment is as essential as the endodontic procedure and depends on patient cooperation. Reinstrumentation of only the affected canal supports structural preservation and fracture resistance, consistent with minimally invasive principles [8]. CBCT provides sensitive detection of periapical changes, detailed internal anatomy assessment, and guidance for selective access, overcoming limitations of two‐dimensional imaging [5, 6, 13, 18]. Despite radiation exposure considerations, CBCT remains essential for SRCR eligibility, while periapical radiographs remain important for initial assessment [4, 6, 19].

In regions with limited access to oral healthcare, patient resistance to tooth‐preserving interventions may influence treatment decisions [18]. During the search process, Brochado‐Martins et al. [20] were identified but excluded from the formal review sample as it did not directly address the purpose of this study. Nevertheless, it offers contextual insight, suggesting that SRCR may be less costly (€2137; range €1944–€2340) and more effective (19.6 years; range 18.3–20.8) than conventional retreatment for a maxillary molar, despite study limitations.

The initial plan for this study was a systematic review, but the diversity of data and study designs in pilot screening made this unfeasible, in line with recommendations for that study design. The mapping of clinical and imaging foundations of SRCR was nonetheless achieved through scoping review and metasynthesis by thematic analysis [9, 10, 11].

Overall, the current evidence is limited by small sample sizes, retrospective designs, lack of control groups, ex vivo methodologies, short observation periods, and evaluation of specific tooth types [6, 7, 8, 12, 13]. This scarcity reflects the novelty of SRCR, despite comprehensive searches across multiple databases and languages. Methodological heterogeneity and limited evidence preclude definitive conclusions, underscoring the need for randomised trials and systematic studies to confirm long‐term efficacy and to explore the impact of systemic patient factors.

Finally, this scoping review formalises early discussion, suggesting promising short‐term outcomes and structural preservation, with CBCT emerging as a central tool for diagnosis, treatment planning, and follow‐up. To the best of our knowledge, this represents the first systematised synthesis of SRCR, providing a structured overview of its clinical and imaging foundations and underscoring areas for further investigation.

## 5 Conclusion

SRCR offers a conservative, potentially effective alternative aligned with minimally invasive endodontic principles, addressing only roots with clinical or imaging evidence of failure. Guided by CBCT, it preserves healthy canals, maintains structural integrity, and may achieve short‐term outcomes comparable to conventional retreatment. Evidence is limited by small samples, retrospective designs, and methodological heterogeneity, highlighting the need for prospective, long‐term studies. This review represents a systematised synthesis of SRCR, integrating clinical and imaging insights to inform future research and clinical decision‐making.

## Author Contributions

**José Evando da Silva‐Filho:** conceptualisation, data curation, formal analysis, investigation, methodology, visualisation, writing – original draft. **Júlia Magalhães‐Saldanha:** data curation, investigation, writing – original draft. **Ana Paula Caracas‐de‐Araújo:** data curation, formal analysis, investigation. **Zildenilson da Silva Sousa:** data curation, investigation, methodology. **Danielle Frota de Albuquerque:** conceptualisation, methodology, supervision, validation; writing – review and editing. **Eduardo Diogo Gurgel‐Filho:** conceptualisation, methodology, project administration, supervision, validation, writing – review and editing. All authors have read and approved the final manuscript.

## Conflicts of Interest

The authors declare no conflicts of interest.

## Supporting information


**File S1:** Database, search strategy, results, selected and included combined. Search Period: 04.18.2025.
**File S2:** Detailed overview of experimental studies.

## Data Availability

The data that supports the findings of this study are available in the Supporting Information of this article.
