# Supplementary material for: Selective Root Canal Retreatment: A Scoping Review and Metasynthesis by Thematic Analysis
Source: Aust Endod J. 2026 Jan 2;52(1):323–30. doi: 10.1111/aej.70050 (PMC13051035; doi:10.1111/aej.70050)
Supplement: Supplementary file 1 — File S1: Database, search strategy, results, selected and included combined. Search Period: 04.18.2025. File S2: Detailed overview of experimental studies. [file AEJ-52-323-s001.docx]

**SUPPLEMENTARY FILES**

**Supplementary File 1.** Database, search strategy, results, selected and included combined. Search Period: 04.18.2025.

| **DATABASE** | **STRATEGY** | **RESULTS** | **SELECTED** | **INCLUDED** |
| --- | --- | --- | --- | --- |
| Cochrane Library | ("Endodontics" OR "Root Canal Therapy") AND "Retreatment" AND (selective OR partial) | 36 (Total):  34 (Trials); 2 (Reviews) | Zero | – |
| Embase | ('endodontics'/exp OR 'root canal therapy'/exp) AND 'retreatment'/exp AND (selective:ti,ab OR partial:ti,ab) | 32 | 4 | Nudera, 2015  Brochado-Martins *et al.*, 2022  Brochado-Martins *et al.*, 2023*  Turky; Elfatah; Hamdy, 2024 |
| PubMed | (("Endodontics"[Mesh] OR "Root Canal Therapy"[Mesh]) AND "Retreatment"[Mesh]) AND ("Selective"[Title/Abstract] OR "Partial"[Title/Abstract]) | 25 | 3 | Nudera, 2015  Brochado-Martins *et al.*, 2022  Turky; Elfatah; Hamdy, 2024 |
| Scopus | TITLE-ABS-KEY ( ( "endodontic retreatment" OR "root canal retreatment" ) AND ( selective OR partial ) ) | 28 | 5 | Nudera, 2015  Nagy; Ghobashy, 2022  Brochado-Martins *et al.*, 2023*  Guerreiro-Viegas; Santos, 2024  Turky; Elfatah; Hamdy, 2024 |
| ScienceDirect | ("endodontic retreatment" OR "root canal retreatment") AND (selective OR partial) | 347 | 1 | Nudera, 2015 |
| Virtual Health Library | ("Endodontia" OR "Tratamento do Canal Radicular" OR "Endodontics" OR "Root Canal Treatment") AND (Retratamento OR "Retratamento Endodôntico" OR Retreatment OR "Endodontic Retreatment") AND (Seletiv* OR Parcial* OR Selectiv* OR Partial*) | 59 (Coleção Completa da “BVS”) | Zero | Brochado-Martins *et al.*, 2022  Brochado-Martins *et al.*, 2023*  Turky; Elfatah; Hamdy, 2024 |
| Web of Science | TS=("endodontic retreatment" OR "root canal retreatment") AND TS=(selective OR partial) | 2 | Zero | – |
|  | Total  (Results with duplicates): | 529 | Total  (Included with no duplicates): | 5 |

*Study excluded due to its focus on cost-effectiveness, which was outside the direct objectives of this review, centered on clinical and imaging foundations.

**Supplementary File 2.** Detailed overview of experimental studies

| **AUTHORSHIP,**  **YEAR AND ORIGIN** | **TITLE** | **OBJECTIVE** | **STUDY TYPE** | **(N)** | **INCL.** | **EXCL.** | **METHODS** | **RESULTS** | **CONCLUSION** |
| --- | --- | --- | --- | --- | --- | --- | --- | --- | --- |
| Brochado-Martins *et al.*, 2022, Netherlands | Outcome of selective root canal retreatment—A retrospective study | (i) To assess the clinical and radiographic/CBCT outcomes of SRCR after ≥12 months; (ii) to analyze the periapical status of untreated roots; (iii) to evaluate tooth survival. | Retrospective study | 75 patients, 75 teeth (195 roots), PR and CBCT groups. All procedures were performed by ESE-certified specialists using DOM. | 1. Multirooted teeth (premolars/molars, maxillary/mandibular).  2. Clinical diagnosis of symptomatic AP and previously treated tooth (AAE, 2009).  3. ≥1 root without lesion and not retreated.  4. Healthy periodontium (≤3 mm probing depth, no mobility or grade I, no bleeding).  5. Intact direct/indirect restorations, no caries or leakage.  6. No signs of fracture/cracks.  7. Pre-, post-, and ≥12-mo follow-up images (PR or CBCT) of diagnostic value. | 1. RCR of all canals.  2. Vertical fracture identified during RCR.  3. Previous surgical endodontic treatment in the root(s).  4. Pre-treatment CBCT without follow-up CBCT.  5. Pre-treatment PR without follow-up PR. | Clinical assessment: symptoms, functional loss, pain, sinus tract, mobility, periodontal condition, restoration quality. Radiographic evaluation via standardized PR/CBCT. Two blinded examiners assessed each root using a 6-point scale (1 to 6). Favorable outcome: scores 4–6 with no clinical signs. The poorest scoring root determined the tooth-level outcome. Discrepancies resolved by consensus. Intra/interobserver agreement: Cohen’s Kappa. Survival: tooth present at follow-up. Prognostic factors analyzed via Fisher’s exact test (α=0.05). | 82 roots retreated; 113 remained untreated. Maxillary molars were most frequently retreated (56%). MB root was most commonly retreated (48.7%), mostly due to missed canals (72.5%). At follow-up, 92.7% of retreated roots had favorable outcomes; 7.3% were unfavorable. Among untreated roots, 3.5% developed new lesions. Tooth-level favorable outcome: 86.7%. No treatment-related factor influenced results (p > .05). High intraobserver agreement (PR: 0.91; CBCT: 0.88). Moderate interobserver agreement (PR: 0.41; CBCT: 0.65). Survival rate: 91.5% (12–48 months). | SRCR achieved favorable outcomes in 86.7% of cases. No significant difference between root- and tooth-level outcomes (p > .05). Untreated roots rarely (3.5%) developed new lesions at follow-up. Survival was 91.5% over 12–48 months. Larger sample and longer follow-up trials are warranted. |
| Nagy; Ghobashy, 2022, Egypt | Selective Root Retreatment: A Randomized Clinical Trial | To compare the prognosis between SRCR and the traditional retreatment approach. | Randomized, controlled, double-blind, multicenter clinical trial with a parallel-group design included | 40 patients with failed root canal treatment, recruited from 2 private endodontic clinics (March 2019–March 2021). | ≥1 root with PAI ≥3, intact restoration. | Systemic disease, multiple root lesions, fractures, crest comm. | 2 arms: selective and traditional. Both used CBCT, DOM, working length with apex locator, ProTaper Next (X1–X3), 2.6% NaOCl, CW obturation. Follow-up: PR at 3, 6, 9, 12 months + CBCT at 12 months. Success defined by PAI (Ørstavik) and complete/incomplete healing. | No differences between groups regarding sex (p = 0.594), age (p = 0.721), or tooth type (p = 0.792). Baseline mean PAI values were similar (p = 0.579). Both groups showed PAI reduction at 12 months, with no significant difference (p = 0.853). | SRCR is an effective minimally invasive option for isolated root disease when remaining roots are healthy. Longer follow-up is recommended. |
| Turky; Elfatah; Hamdy, 2024, Egypt | Does selective root canal retreatment preserve the tooth’s fracture resistance? An ex-vivo study | To assess fracture resistance following SRCR versus conventional approach. | *Ex Vivo* Study | 33 extracted teeth. | Mandibular first molars with mature roots, canal curvatures <20°, radius <5 mm, extracted for periodontal reasons. | Teeth with restorations, caries, resorptions, non-negotiable canals, or cracks (20X) after 3D matching. | Teeth cleaned, immersed in 5.25% NaOCl (30 min), stored in 0.1% thymol. Mounted in acrylic with simulated PDL. MOD cavities prepared. Endodontics: HyFlex CM, 5.25% NaOCl, 17% EDTA, single-cone + BC sealer. Restoration: bulk-fill + conventional composite. Aging: 75k cycles + 600 thermocycles. Groups: control (n=11), full retreatment, selective (distal root). New restorations applied. Fracture tested (Instron, 1 mm/min). Failure type via USB microscope (35X). ANOVA (α=0.05). | Fracture resistance was lower in the Conventional-NSR group (867.7 ± 108.9 N) compared to the Selective-NSR group (1106.8 ± 159.8 N) (P = 0.012). Both NSR groups showed lower values than the control (1337.7 ± 250.5 N). Non-repairable fractures were more frequent in the Conventional-NSR group (54.5%) than in the Selective (36.4%) and Control (18.2%) groups. | SRCR appears to be a promising alternative to conventional NSR, with potential to preserve fracture resistance in endodontically treated multirooted teeth. |

AAE, American Association of Endodontists; AP, Apical Periodontitis; BC, Bioceramic; CBCT, Cone-Beam Computed Tomography; CW, Continuous Wave; DOM, Dental Operating Microscope; EDTA, Ethylenediaminetetraacetic Acid; ESE, European Society of Endodontology; f/u, Follow-Up; MB, Mesiobuccal; MOD, Mesio-Occluso-Distal; NaOCl, Sodium Hypochlorite; NSR, Non-Selective Retreatment; PAI, Periapical Index; PDL, Periodontal Ligament; PR, Periapical Radiograph; RCR, Root Canal Retreatment; S, Selective; SRCR, Selective Root Canal Retreatment; T, Traditional; WL, Working Length; X1–X3, ProTaper Next Rotary Files (X1–X3).
